# Treatment and outcomes of an Australian cohort of outpatients with bipolar I or schizoaffective disorder over twenty-four months: implications for clinical practice

**DOI:** 10.1186/1471-244X-12-228

**Published:** 2012-12-17

**Authors:** Jayashri Kulkarni, Sacha Filia, Lesley Berk, Kate Filia, Seetal Dodd, Anthony de Castella, Alan JM Brnabic, Amanda J Lowry, Katarina Kelin, William Montgomery, Paul B Fitzgerald, Michael Berk

**Affiliations:** 1 Monash Alfred Psychiatry Research Centre, The Alfred Hospital and Monash University, Central Clinical School, 607 St Kilda Rd, Melbourne, VIC, 3004, Australia; 2 Department of Psychiatry, The University of Melbourne, Parkville, VIC, 3010, Australia; 3 Optum, Lilyfield, NSW, 2040, Australia; 4 Eli Lilly Australia Pty Ltd, 112 Wharf Road, West Ryde, NSW, 2114, Australia; 5 Global Health Outcomes, Intercontinental Region, Eli Lilly Australia Pty Ltd, 112 Wharf Road, West Ryde, NSW, 2114, Australia; 6 Orygen Research Centre, Parkville, VIC, 3052, Australia; 7 Florey Institute for Neuroscience and Mental Health, University of Melbourne, Parkville, 3010, Victoria, Australia; 8 School of Medicine, Deakin University, Geelong, VIC, 3220, Australia

## Abstract

**Background:**

The Bipolar Comprehensive Outcomes Study (BCOS) is a 2-year, prospective, non-interventional, observational study designed to explore the clinical and functional outcomes associated with ‘real-world’ treatment of participants with bipolar I or schizoaffective disorder. All participants received treatment as usual. There was no study medication.

**Methods:**

Participants prescribed either conventional mood stabilizers (CMS; n = 155) alone, or olanzapine with, or without, CMS (olanzapine ± CMS; n = 84) were assessed every 3 months using several measures, including the Young Mania Rating Scale, 21-item Hamilton Depression Rating Scale, Clinical Global Impressions Scale – Bipolar Version, and the EuroQol Instrument. This paper reports 24-month longitudinal clinical, pharmacological, functional, and socioeconomic data.

**Results:**

On average, participants were 42 (range 18 to 79) years of age, 58%; were female, and 73%; had a diagnosis of bipolar I. Polypharmacy was the usual approach to pharmacological treatment; participants took a median of 5 different psychotropic medications over the course of the study, and spent a median proportion of time of 100%; of the study on mood stabilizers, 90%; on antipsychotics, 9%; on antidepressants, and 5%; on benzodiazepines/hypnotics. By 24 months, the majority of participants had achieved both symptomatic and syndromal remission of both mania and depression. Symptomatic relapse rates were similar for both the CMS alone (65%;) and the olanzapine ± CMS (61%;) cohorts.

**Conclusions:**

Participants with bipolar I or schizoaffective disorder in this study were receiving complex medication treatments that were often discordant with recommendations made in contemporary major treatment guidelines. The majority of study participants demonstrated some clinical and functional improvements, but not all achieved remission of symptoms or syndrome.

## Background

Bipolar disorder (BD) is a complex and severe mental illness, with a fluctuating course, characterized by acute, affective episodes with full or partial inter-episode remissions. Adding to the complexity of BD is the “mixed” affective syndrome, in which the person experiences both depressive and manic symptoms simultaneously [1]. The lifetime prevalence of BD I (manic, mixed or depressed) and BD II (hypomanic, depressed) is estimated as 2.1%; of the US population, however, if one considers sub-threshold cases, the lifetime prevalence may be as high as 4.5%; [2]. Similar figures were reported by a recent Australian-based study in which the prevalence of BD was estimated between 2.5%; and 2.9%; [3,4].

The global burden of BD is significant as it represents the sixth leading cause of years lived with disability [5]. Individuals with BD often also suffer from comorbid psychological and medical issues and are more likely to commit suicide [6]. A recent US-based survey found that many respondents with BD also had histories of anxiety (75%;), impulse-control (63%;) and/or substance use (42%;) disorders; with 70%; of these respondents experiencing ≥3 disorders [2].

Due to the varying mood-states and different expressions of the illness, management of BD is complicated. For decades, lithium was considered the cornerstone of pharmacologic treatment for BD [7] and remains a first line treatment in most major guidelines [8,9]. However, an increasingly diverse range of pharmacological agents (i.e., anticonvulsants, antidepressants and antipsychotics) has found a place in the treatment of BD at different stages of the illness [10-15]. Surveys of prescribed medicines used for the treatment of BD have consistently described complex treatment regimens, with polypharmacy being the norm rather than the exception [16].

Several large observational studies of patients with BD and schizoaffective disorder (SAD) have been conducted and subsequently published [17-24]. Among them, the Bipolar Comprehensive Outcomes Study (BCOS) is a 2-year, prospective, non-interventional, observational study designed with the purpose of improving the understanding of treatment and outcomes of patients diagnosed with BD I or SAD [21-24]. The rationale for studying these two patient populations concomitantly is that both SAD and BD share common features as well as treatment approaches [25,26]. The BCOS was designed to provide a comprehensive assessment of outcomes. Several key benefits of the BCOS are: a) it is the first prospective observational study conducted in Australia assessing this patient population; b) detailed information on clinical status and medications taken (including validated sources of medications prescribed) were collected; and c) the study design allowed for information from many data sources to be integrated (i.e., case report form data, national health insurance claims, and hospital and community data).

The observational design was chosen for the BCOS to complement information available from other study designs, such as randomized controlled trials (RCTs). While RCTs have higher internal validity, observational studies offer several advantages over RCTs including: a) lower cost; b) increased timeliness; c) fewer inclusion and exclusion criteria, thus providing an opportunity to study a more diverse patient population over longer periods of time in a naturalistic setting; and most importantly, d) the ability to identify risk factors and prognostic indicators in situations which would be impossible or unethical for RCTs [25,27-30]. With a complex condition like BD, it was hoped that the observational design would help to achieve the study objectives.

The broad objective of BCOS was to better understand the long-term clinical, functional, social and economic outcomes achieved by participants being treated for BD I or SAD. As part of this objective, we aimed to evaluate current clinical practices, including pharmacological treatment. Specifically, we were interested in examining the 2-year outcomes for participants following treatment with either conventional mood stabilizers (CMS) alone, or olanzapine with, or without, CMS (olanzapine ± CMS).

## Methods

### Study design

The BCOS was a 2-year, prospective, non-interventional, observational study of outpatients prescribed CMS with or without olanzapine for the treatment of BD I or SAD (study code F1D-AY-B004). All participants provided informed consent to participate in the study, which was conducted in accordance with Australian ethics and privacy laws. Detailed information about the study design, patient populations and methodology has been previously published [22]. In short, the BCOS was conducted at two sites in Victoria, Australia: the Monash Alfred Psychiatry Research Centre at the Alfred and Monash University in Melbourne, and the Barwon Psychiatric Research Unit in Geelong. This study included consenting males and females at least 18 years of age, with a primary diagnosis of BD I (manic, mixed, or depressed episode) or SAD as defined by the *Diagnostic and Statistical Manual of Mental Disorders, Fourth Edition Text Revision* (DSM-IV-TR) [31], and their diagnosis was confirmed by the Mini-International Neuropsychiatric Review (MINI) Version 5 [32]. In addition, participants had to be prescribed at least one of the following CMS (lithium carbonate, sodium valproate, or carbamazepine) or olanzapine as a mood stabilizer, or a combination of CMS plus olanzapine, actively participated in the interview process, and were willing and able to complete the self-administered instruments. In order to obtain a representative sample of participants, exclusion criteria were minimal, only those participants: with a DSM-IV-TR diagnosis of schizophrenia, organic psychosis or dementia; involved in a controlled clinical trial 30 days prior to the study or at any time during the study; who did not meet all of the inclusion criteria; were considered ineligible. Trained evaluators assessed participants on 9 separate occasions – at study entry, and every 3 months up to 24 months. Data were obtained from participant interviews, patient medical records, and electronic administrative health care records, and were incorporated into a running record. Information collected included quality of life (both subjective and objective measures), use of concomitant medications, stability of treatment, medication adherence (patient self-reported), employment status, and any lost productivity due to illness. This study was not designed to assess safety; all adverse events were reported to the Adverse Drug Reactions Advisory Committee in accordance with Australian safety requirements.

### Treatment

Since this was an observational study, participants were not randomized to treatment, but instead, were maintained on their prior medication regimen. All treatment decisions were made by the participant’s primary treating clinicians, independent of the study. Participants were recruited (from September 2003 to November 2005) into one of two cohorts: a) those who were receiving at least one of the listed CMS, excluding olanzapine (i.e., CMS alone); or b) those receiving, olanzapine as a mood stabilizer alone, or in combination, with at least one of the listed CMS (i.e., olanzapine ± CMS), at study entry. Selection of the pre-specified treatments included in this study was based on the approved indication and frequency of use of each of these treatments in Australia at that time. Participants with BD I were not recruited into the olanzapine ± CMS cohort until the Therapeutic Goods Administration granted approval for the use of olanzapine for preventing the recurrence of manic, mixed, or depressive episodes in BD I in October 2003.

### Measures and definitions

Clinician and study participant self-rated measures were used to capture a range of clinical, social and functional outcomes associated with treatment throughout the 2-year observation period. The clinical status of each participant was assessed using the following scales: a) Clinical Global Impressions-Bipolar Version Severity of Illness scale (CGI-BP) [33]; b) Young Mania Rating Scale (YMRS) [34]; and c) 21-item Hamilton Depression Rating scale (HAMD_21_) [35].

Symptomatic remission from a manic state was defined as a YMRS total score of ≤12 [36] and HAMD_21_ total score of ≤8; from a depressive state, remission was defined as a HAMD_21_ total score of ≤8 (based on the consensus definition proposed by the International Society for Bipolar Disorders task force, assuming the equivalence of HAMD_21_ ≤ 8 and HAMD_17_ < 5 or <7 [37]). Relapse rates were only assessed in those participants that achieved symptomatic remission as defined previously. Symptomatic relapse to a manic state was defined as a YMRS total score of ≥15 in only those participants who met the criteria for symptomatic remission of mania. Relapse to a depressed state was defined as a HAMD_21_ total score of ≥15 in only those participants who met the criteria for symptomatic remission of depression. A psychiatric hospital admission also defined a symptomatic relapse in those participants who previously established symptomatic remission.

Definitions of syndromal remission were modified from those used in the McLean first-episode psychosis project [38]. Syndromal remission from a manic state was defined as all DSM-IV-TR ‘A’ and ‘B’ criteria for current manic episode being no worse than mild (≤3 on a 1 to 7 scale), and no more than two ‘B’ criteria rated as mild (=3 on a 1 to 7 scale). Syndromal remission from a depressive state was defined as all DSM-IV-TR ‘A’ criteria for current major depressive episode no worse than mild (≤3 on a 1 to 7 scale), and no more than three ‘A’ criteria rated as mild (=3 on a 1 to 7 scale). Relapse rates were only assessed in those participants who achieved syndromal remission as defined previously. Syndromal relapse to a manic state was defined as meeting DSM-IV-TR criteria for a current manic episode in only those participants who previously fulfilled the criteria for syndromal remission of mania. Relapse to a depressed state was defined as meeting DSM-IV-TR criteria for a current depressive episode in only those participants who first met the criteria for syndromal remission for depression.

The EuroQol instrument (EQ-5D) [39] and 36-item Short-Form Health Survey (SF-36) [40] were used to capture self-reported health-related quality of life. The social functioning of participants was evaluated using the Streamlined Longitudinal Interview Clinical Evaluation from the Longitudinal Interval Follow-up Evaluation (SLICE/LIFE) [41], and the Diagnostic Interview for Psychosis (DIP) [42] was used to rate impairment in work, housework, and study. Medical history and sociodemographic information were collected at study entry; key items such as employment status and suicidality were collected throughout the study. Information about pharmacological treatments taken, including dosage, start and stop dates, previous medication history, stability of treatment and adherence (patient self-reported) was collected at each visit for all mood stabilizers, antipsychotics, and antidepressants. Concomitant use of psychotropic medications was also captured.

### Statistical analysis

In order to fully explore the relationship between treatment and outcomes, two statistical analysis approaches were used to assign participants to cohorts. These approaches were based on either a) the medication participants were receiving at study entry (i.e., CMS-alone or olanzapine ± CMS); or b) the predominant treatment (PT) the participant received during the study (i.e., PT-CMS or PT-olanzapine). For each participant, PT was defined as the drug with the highest cumulative number of defined daily dose (DDD) units [43] over the course of the study. In cases where a medication dose was missing, a value of 1 DDD unit was assigned. Where the outcome was remission, the PT period was calculated from the study entry visit to the first remission visit. Where relapse was the outcome, the PT period was calculated from the first remission visit to the first relapse visit. For the purpose of analysis, the remission visit was considered as the new study entry for this period.

All statistical analyses were performed using SAS® for Windows, version 9.1.3 (SAS Institute, Cary, NC, USA). Descriptive statistics were used to characterize participants at study entry for all demographic and clinical measures. Propensity scores were constructed and used in an attempt to create balance between comparison groups [44,45]. Variables in the propensity score model included race, gender, income, diagnosis, age, body mass index, recurrent major depressive episode (MDE), alcohol abuse risk, length of hospitalization, medication adherence, attempted suicide (in the past month), amount of time treated with either mood stabilizers, antipsychotics, antidepressants, or benzodiazepines/hypnotics since the previous visit, episode(s) of mania or hypomania in the past (based on MINI), EQ-5D and SF-36 summary scores, and total YMRS, HAMD_21_, and SLICE/LIFE scores.

All adjusted proportions and Cox-regression models were controlled for propensity score as well as several additional prespecified covariates. The covariates assessed at study entry were age, gender, diagnosis (BD I or SAD), partner status (from SLICE/LIFE), employment status, smoking status, attempted suicide in the month prior to study entry (based on MINI), recurrent MDE (based on MINI), alcohol abuse risk and CGI-BP total score. Other covariates included site, hospitalized at visit, length of hospitalization, alcohol dependence in previous year (based on MINI), past episode(s) of mania (based on MINI), medication adherence, and amount of time treated with either mood stabilizers, benzodiazepines/hypnotics, antipsychotics, or antidepressants since the previous visit.

The proportions of participants who either relapsed or went in to remission were reported with and without adjustment. Adjusted proportions were implemented using generalized linear mixed effects models. Median time to relapse after remission was calculated from adjusted Cox regression models with bootstrapped 95%; confidence intervals (CI).

Longitudinal profiles of quality of life (as measured by SF-36) were examined using mixed models repeated measures. The model included the intercept and time as random effects, with the remaining variables (listed above as additional covariates) treated as fixed effects. The Spatial Power covariance matrix was used to estimate within-participant errors, and the Kenward-Roger method was used to estimate denominator degrees of freedom. In addition, the Type III sum-of-squares was used for the least-squares means.

## Results

### Participant characteristics

**Figure 1:**
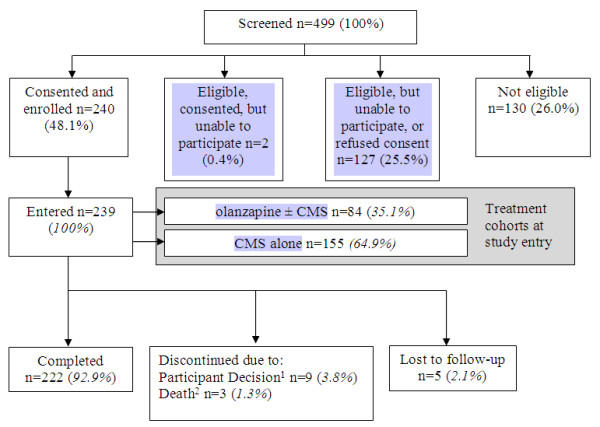
**Participant Disposition.**^1^ One participant withdrew consent after the first study visit; study entry results n = 239, post-study entry n = 238 (unless otherwise stated). ^2^ Two participants died due to natural causes, one from suicide.

Of a total of 499 participants screened, 48%; (n = 240) were enrolled, 26%; (n = 129) were eligible, but unable to participate or refused consent, and 26%; (n = 130) did not meet the inclusion criteria (Figure 1). Of the 239 participants who had post-study entry visits 222 (93%;) completed the study.

Overall, participants in BCOS were on average 42 years of age and first exhibited symptoms of mental illness at age 18 (Table 1). The majority of patients met DSM-IV-TR criteria for BD I (73.2%; [175/239] vs. 26.8%; [64/239] for SAD). Clinical status at study entry was similar for all participants, with the exception of HAMD_21_ scores, which were significantly (p < 0.05) higher for participants with SAD (Table 1). In general, participants with SAD reported more occupational and social dysfunction at study entry than those with BD I (Table 1).

**Table 1:** Participant population characteristics at study entry

**Characteristic**	**SAD**	**BDI**	**All**
	**n = 64**	**n = 175**	**N = 239**
*Demographics*			
Age, mean (range), years	39.6 (20–66)	42.6 (19–79)	41.8 (18–79)
Gender, women,%;	50.0%;	61.1%;	58.3%;
*Clinical status*			
Age at first symptoms of mental illness, median (range), years	18 (5–43)	17 (3–65)	17.5 (3–65)
CGI-BP-S overall score, mean (SD)	3.7 (1.2)	3.9 (1.4)	3.8 (1.3)
YMRS total score, median (range)	4.5 (0–39)	5.0 (0–45)	5.0 (0–45)
HAMD_21_ total score, mean (SD)	15.5 (8.4)	12.7 (8.5)	13.4 (8.6)*
At least 1 hospital admission; past 3 months,%;	35.9%;	32.0%;	33.1%;
*Functional status*			
EQ-5D health state score, mean (SD)	61.6 (22.7)	68.2 (18.8)	66.4 (20.1)*
Daily smoker,%;	56.3%;	49.1%;	51.0%;
Daily alcohol consumption; past 3 months,%;	4.7%;	13.1%;	10.9%;
Alcohol dependence; past 12 months,%;	14.1%;	18.3%;	17.2%;
Unemployed,%;	48.4%;	22.3%;	29.3%;*
Employed/studying/housework,%;	43.8%;	68.6%;	61.9%;*
Have a partner,%;	26.6%;	46.9%;	41.4%;*
Considered suicide in the past month,%;	75.0%;	58.9%;	63.2%;*

Abbreviations: BD = bipolar I disorder; CGI-BP-S = Clinical Global Impressions-Bipolar Version – Severity of Illness scale (range 1–7); EQ-5D = EuroQol instrument; HAMD_21_ = 21-item Hamilton Depression Rating scale; SAD = schizoaffective disorder; SD = standard deviation; YMRS = Young Mania Rating Scale. *Indicates statistical significance (p < 0.05) for comparison of BD and SAD participants. All p values are based on Fisher’s exact test except for HAMD_21_ and EQ-5D scores, which were tested using F-test analysis of variance.

Although the participant population as a whole exhibited sub-threshold hypomanic features, 19.0%; of participants were manic at study entry (YMRS score ≥15). Approximately one-quarter (25.4%;) of study participants were suffering moderate-severe depression (HAMD_21_ score ≥19; [46]) when they entered the study; for 68.3%; (n = 41) of these participants, their depression had improved to a mild or moderate state (HAMD_21_ score <19; [46]) by 24 months.

Forty-four percent (n = 105) of participants were admitted to hospital at least once during the study. Of those hospitalized, 43.8%; (n = 46) were single admissions, although the median length of stay was 21 days (range 1 to 345). For multiple admissions, 26.7%; of participants (n = 28) were admitted twice, 7.6%; (n = 8) had three admissions, 6.7%; (n = 7) had four admissions, and 5.7%; (n = 6) had more than four (up to 9) admissions during the study period.

### Symptomatic relapse

One of the study’s objectives was to compare the proportion of participants who experienced symptomatic relapse to either manic or depressive states following treatment with either CMS alone (n = 155), or olanzapine ± CMS (n = 84) (at study entry). Using this analysis approach, there were no statistically significant differences between the treatment groups: 60.7%; (n = 37) participants met symptomatic relapse criteria in the olanzapine ± CMS group compared with 65%; (n = 76) participants in the CMS alone group (p = 0.62). Even after adjustment with propensity scores and preselected covariates, there was no significant difference between the two treatment groups (proportion of participants [95%; CI], olanzapine ± CMS versus CMS alone, 39.2%; [15.5%; to 69.5%;] vs 59.8%; [33.3%; to 81.6%;]).

In order to better define the treatment groups, medication patterns (including dosage) were examined for each participant, and their PT (based on DDD units) was identified. The results based on these PT cohorts were similar to those observed with the ‘prescribed at study entry’ groups using adjusted relapse rates (proportion of participants [95%; CI], PT-olanzapine [n = 57] versus PT-CMS [n = 121], 46.8%; [19.1%; to 76.6%;] vs 56.9%; [30.6%; to 79.9%;]; hazard ratio 0.765 [0.425 to 1.378]). The median time (calculated from the Cox model) to relapse was 286 days (182 to 624 days) for the PT-olanzapine cohort compared with 230 days (182 to 357 days) for the PT-CMS cohort.

### Symptomatic and syndromal remission and relapse

**Figure 2:**
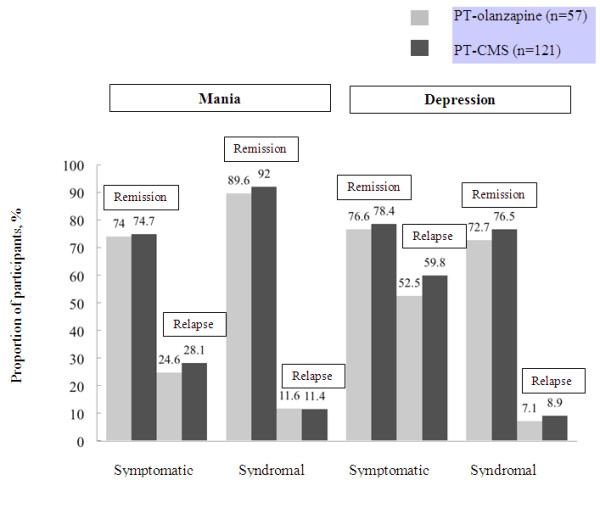
**Proportion of Participants Experiencing Symptomatic**^**1 **^**or Syndromal**^**2 **^**Remission or Relapse into Mania or Depression.** These are unadjusted data based on predominant treatment. Refer to Methods for the definitions of symptomatic relapse, symptomatic remission, syndromal relapse and syndromal remission for mania and depression. Groups were defined based on predominant treatment (PT; based on DDD units). PT-olanzapine refers to those participants (n = 57) in which olanzapine had the highest DDD unit value. PT-CMS refers to those participants (n = 121) in which a CMS had the highest DDD unit value. ^1^Symptomatic remission: from a manic state is defined as a YMRS total score of ≤12 and HAMD_21_ ≤8; from a depressive state is defined as a HAMD_21_ total score of ≤8. Symptomatic relapse: to a manic state, is based on a YMRS total score of ≥15 after having met the criteria for symptomatic remission (mania); to a depressed state is based on a HAMD_21_ total score of ≥15 after having met the criterion for symptomatic remission (depression). ^2^Syndromal remission: from a manic state is defined as all DSM-IV-TR ‘A’ and ‘B’ criteria for current manic episode are no worse than mild (≤3 on a 1 to 7 scale), and no more than two ‘B’ criteria are mild (=3 on a 1–7 scale); from a depressive state is defined as all DSM-IV-TR ‘A’ criteria for current major depressive episode are no worse than mild (≤3 on a 1–7 scale), and no more than three ‘A’ criteria are mild (=3 on a 1–7 scale). Syndromal relapse: to a manic state is based on meeting DSM-IV-TR criteria for current manic episode after having met the criteria for syndromal remission (mania); to a depressed state is based on meeting DSM-IV-TR criteria for current depressive episode after having met the criteria for syndromal remission (depression).

To explore clinical outcomes more closely, we examined symptomatic and syndromal remission and relapse (as defined in the methods) to either depression or mania as separate events for participants receiving either olanzapine or CMS as the PT (Figure 2). The same analysis was conducted on the ‘prescribed at study entry’ cohorts; the findings were concordant with those shown in Figure 2.

By 24 months, the majority of participants in both the PT-olanzapine (n = 57) and PT-CMS (n = 121) groups had ever achieved both symptomatic and syndromal remission of both mania and depression (Figure 2). Regardless of PT, the overall symptomatic remission rates were similar for mania and depression (74.5%; vs 77.8%;, respectively). Symptomatic relapse to a depressed state was more than twice as common as relapse into a manic state (pooled averages of 57.5%; vs 27.0%;, respectively). Syndromal relapse (pooled averages of 8.3%; and 11.5%; for depression and mania, respectively) was less frequent than symptomatic relapse. There were no statistically significant differences observed between the treatment cohorts.

### Treatment patterns

**Figure 3:**
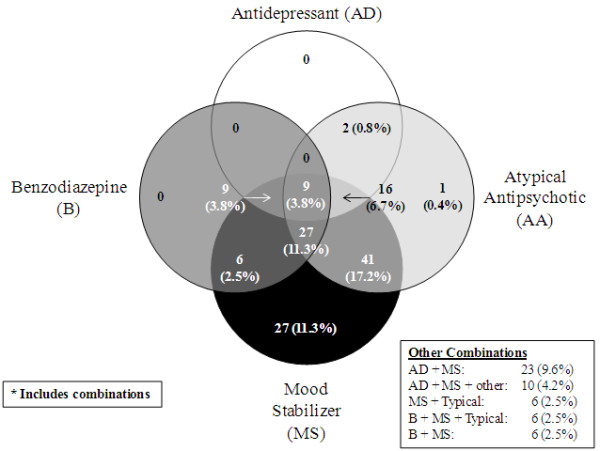
**Patterns of Psychotropic Medications**^**1 **^**Taken at First Visit.**^1^ Psychotropic medications included are: atypical antipsychotics (AA), antidepressants (AD), benzodiazepines (B), mood stabilizers (MS; olanzapine, lithium, valporate, and carbemazepine), and other (i.e., anticonvulsants, anticholinergics, anxiolytics, and hypnotics).

**Figure 4:**
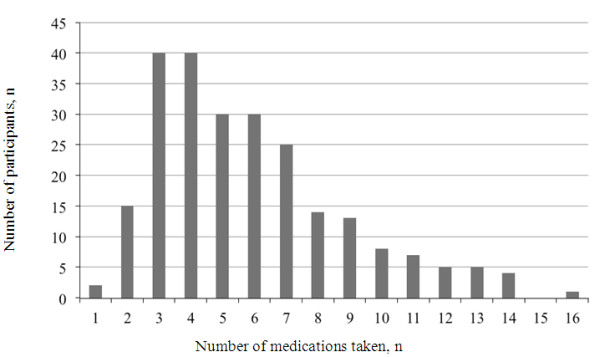
**Treatment Patterns: Total Number of Psychotropic Medications**^**1 **^**Taken During the Study.**^1^ Psychotropic medications included are: antipsychotics, antidepressants, benzodiazepines, mood stabilizers (olanzapine, lithium, valporate, and carbemazepine), anticonvulsants (lamotrigine), anticholinergics, anxiolytics, and hypnotics.

Analysis of prescription pattern data suggests that all BCOS participants received some form of mood stabilizer consistently over the course of the study. Initially, participants were most commonly prescribed an atypical antipsychotic in combination with a CMS at study entry (17.2%;), followed by mood stabilizers (11.3%;), atypical antipsychotics/CMS/benzodiazapines (11.3%;), then antidepressants/CMS (9.6%;), although multiple combinations of medications across classes were used (Figure 3). Irrespective of the study entry treatment group, polypharmacy appeared to be the usual approach to pharmacological treatment for BCOS participants, with multiple medications taken at each study visit (Figure 4). Participants took a median of 5 different medications (ranging from 1 to 16) over the course of the study. Please note that, in Figures 3 and 4, olanzapine is grouped with CMS in a broader ‘mood stabilizer’ grouping, which differs from the CMS grouping used in other analyses.

Medication regimens taken by BCOS participants were complex. Most participants spent a variable amount of time on different medications; the median proportion of time (and range) spent on medication from study entry to 24 months was 100.0%; (0%; to 272.5%;) for mood stabilizers, 90.4%; (0%; to 330.9%;) for antipsychotics, 9.0%; (0%; to 200.0%;) for antidepressants, and 4.5%; (0%; to 281.5%;) for benzodiazepines/hypnotics. Treatment times exceeding 100%; are due to participants receiving more than one medication from the same drug class. Similar treatment durations were also observed in the first 12 months of the study (median time spent on medication [range]); 100%; [0%; to 263.1%;] for mood stabilizers, 96.7%; [0%; to 359.0%;] for antipsychotics, 6.1%; [0%; to 200.0%;] for antidepressants, and 1.1%; [0%; to 263.7%;] for benzodiazepines/hypnotics.

### Health related quality of life and other patient reported outcomes

Despite the majority of participants experiencing mild depressive and manic symptoms at study entry, study participants reported considerable functional impairment and comorbidities. Approximately 40%; of participants were in a permanent relationship, and 63%; had considered suicide in the previous month (Table 1). Further, about half (51%;) the participants smoked daily, 11%; consumed alcohol daily, with 17%; of participants meeting the criteria for alcohol dependence in the year prior to joining the study (Table 1). Of the 41.6%; (n = 99) of participants who were employed (full or part-time) at study entry, 29.3%; (n = 29) were no longer in paid employment by the end of the study, and 26.6%; (n = 37) of participants without paid employment at study entry had secured full or part-time jobs by 24 months. Compared with study entry, unemployment decreased at 24 months (from 29.3%; to 23.1%;), although it remained higher than the Australia-wide rate of 5.4%; [47].

**Figure 5:**
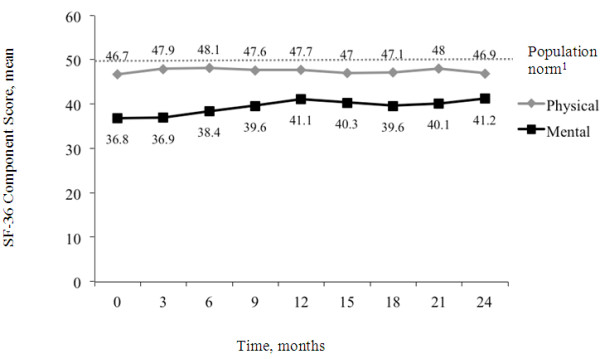
**Mental and Physical Health Assessment at Each Visit During the 24-Month Study.** Mental and Physical health were assessed using the Short-Form Health Survey (SF-36). ^1^ Population norm as provided by the SF-36 organization http://www.sf-36.org/tools/SF36.shtml.

Overall, participants experienced some improvements in quality of life during the course of the study. The SF-36 mental health component scores improved significantly over 24 months (from 36.8 to 41.2; p = 0.029; Figure 5), although physical health component scores remained consistent across the study (from 46.7 to 46.9; p = 0.384).

## Discussion

As a longitudinal, prospective, observational study, the BCOS captured information on the course of treatment of participants with BD I and SAD in a naturalistic setting, and examined a broad range of study participant outcomes over a 2-year period. One in two participants screened for the study were not enrolled, due to a combination of ineligibility (26%;) and refusal of consent or being unable to participate (26%;); however, post-enrollment participant retention was very high (93%;). Unlike those in more restrictive RCT settings, BCOS participants were not excluded based on comorbidities, and were free to use any adjunctive medications or therapies. However, the requirement to be receiving at least one of the pre-specified agents as a mood stabilizer at study entry excluded patients receiving other treatment regimens.

In considering the clinical correlates of BD, we found high symptomatic remission rates for mania and depression (>74%;), demonstrating that participants experienced adequate symptom control across both poles during the study. However, the high relapse rate for depression (58%; vs. 27%; for manic relapse) suggests that maintaining long-term management of depressive symptoms is more difficult to achieve. This aligns with results from a long-term follow up study of BD I patients, in which patients were symptomatically ill 47%; of the time, with depressive symptoms predominating [48].

A major finding from this study was the extent of polypharmacy used in treating people with BD. The pre-specified comparison between the symptomatic relapse rate of participants prescribed olanzapine ± CMS or CMS alone at study entry was confounded by the complexity of the treatment that participants received over time. Polypharmacy is also prevalent in other countries; a large US study of patients with BD (n = 7406), revealed that, although 33%; of patients received an initial prescription for more than one psychotropic agent, 12 months later, the polypharmacy rate among those still receiving treatment had risen to 50%; [49].

The key finding with respect to social outcomes and quality of life was that while unemployment rates improved over the 24-month study period, the rate was still higher than in the general Australian population [50]. In addition, participants described overall satisfaction with life and, although their mental states improved over time, their physical health did not. It should be noted, however, that the physical health of this study population was quite good, with SF-36 physical health state scores within one standard deviation of the norm. Examination of personal relationships showed that a significant proportion of people in this study with BD did not have a meaningful, close relationship.

As mentioned previously, observational studies offer several advantages over RCTs including: a) the opportunity to study a more diverse patient population, over longer periods of time and; b) the ability to identify risk factors and prognostic indicators in situations in which it would be impossible or unethical for RCTs to do so [25,27-30]. Specific to the BCOS, strengths of this study included high external validity, a patient centric approach, high retention rate, real world setting to assess treatment effectiveness, and the comprehensive use of functional and quality of life scales. Despite the strengths of the BCOS, several limitations should be considered when interpreting these data. First, observational studies are not designed to establish causal relationships but rather to examine associations; these data should be interpreted with caution as not all comparisons were powered *a priori*. Second, the participants were required to provide consent at study entry, thus excluding many of the most severely ill patients, although 19%; of participants in this study were manic and 25.4%; were suffering severe depression (HAMD_21_ score ≥19; [46]). Third, concomitant therapies such as counselling and psychotherapy were not captured, thus their potential impact on outcomes could not be evaluated. Fourth, HAMD_21_ and YMRS scores assessed symptoms in the week prior to the assessment visit. Given the fluctuating course of symptoms, this week may not adequately reflect symptoms across the 3-month visit interval. However, the 2-year prospective observation period allowed an extended period of observation, so that the fluctuating course of illness and outcomes could be captured. Finally, the lack of randomization exposes a potential for selection bias and unmeasured confounding; attempts were made to address potential selection bias by adjusting the analysis for known confounding using propensity scores, although unmeasured confounding remains a limitation. For example, participants were grouped irrespective of diagnosis; the BCOS design pre-specified approximately equal recruitment into each of the two primary treatment cohorts (olanzapine ± CMS and CMS alone), however, more participants with a diagnosis of SAD were in the olanzapine ± CMS cohort (38%;) as compared with the CMS alone cohort (22%;); since the BD indication was new for olanzapine, these participants may not reflect those currently using this medication.

Our naturalistic data suggest that participants were receiving diverse, complex medication treatments that led to some overall clinical and functional improvement. However, not all participants achieved remission of symptoms or syndrome. Persistent depressive symptoms were common for this sample, with 58%; of participants experiencing symptomatic relapse of depression at some point. Given the complexity of treatment, we split participants into PT cohorts to explore the comparative treatment effectiveness but with limited success. Few differences were observed between the treatment cohorts; the use of multiple mood stabilizers and other concomitant medications made it difficult to attribute reported outcomes to any one treatment group, and thus elucidate the role of individual medications. This is an important limitation, particularly in terms of making recommendations on prescribing practices. It should be noted that a recent multiple-treatment meta-analysis (data from 68 RCTs) which ranked efficacy and acceptability of drugs used to treat mania concluded that olanzapine was one of two antipsychotic drugs that ranked superior in terms of efficacy and acceptability [11,51]. An analysis of direct and indirect costs borne by the BCOS participant population, including healthcare resource use and lost productivity, will be examined in a separate publication.

## Conclusions

The findings from the BCOS describe a complex patient population receiving treatment regimens that were often discordant with recommendations made in the major treatment guidelines for either BD or SAD. The polypharmacy approach used to treat many of the study participants was associated with some improvement in clinical and functional outcomes. However, this raises a series of questions regarding the pragmatic utility of current clinical guidelines [51]. The abundance and diversity of treatments reported in the BCOS reflect the complexity of these disorders and suggests that therapy should be tailored to the unique requirements of each patient.

## Competing interests

Funding for this study was provided by Eli Lilly Australia. The chief study investigators (J. Kulkarni and M. Berk) are independent academic clinicians and directed the study design and conduct. Lilly Medical employees were involved in study design, analysis and interpretation of data, and in the writing of the report. The Lilly authors listed (A. Brnabic, W. Montgomery, K. Kelin and A.J. Lowry) participated in the decision to submit the paper for publication. Lilly had no role in data collection.

J. Kulkarni, P.B. Fitzgerald, A. de Castella, and S. Dodd have received research funding and/or speaker's fees and/or funding to attend conferences from Eli Lilly. M. Berk has received grant/research support from the Stanley Medical Research Foundation, MBF, NHMRC, Beyond Blue, Geelong Medical Research Foundation, Bristol Myers Squibb, Eli Lilly, Glaxo SmithKline, Organon, Novartis, Mayne Pharma and Servier; has been a speaker for Astra Zeneca, Bristol Myers Squibb, Eli Lilly, Glaxo SmithKline, Janssen Cilag, Lundbeck, Pfizer, Sanofi Synthelabo, Servier, Solvay, Wyeth and acted as a consultant for Astra Zeneca, Bristol Myers Squibb, Eli Lilly, Glaxo SmithKline, Janssen Cilag, Lundbeck, and Servier. L. Berk is supported by an NHMRC scholarship. W. Montgomery, K. Kelin, and A.J. Lowry are employees of Eli Lilly Australia. A. Brnabic was employed by Eli Lilly Australia at the time of manuscript development.

## Authors' contributions

AB was involved in study design, statistical planning, analysis, interpretation and presentation of results. LB was involved in conducting the assessments with participants and editing the manuscript. MB was a principle investigator on the study and was involved in editing the manuscript. AD was involved in the design and conduct of the study, and has contributed to the analysis and presentation of results. AL was involved in the data interpretation and drafting of the manuscript. KF was involved in the project coordination and data collection for this study, and contributed to the overall study design and preparation and review of this manuscript. SF was involved in the project coordination and data collection for this study, and contributed to the overall study design and preparation and review of this manuscript. JK was a principle investigator on the study and was involved in editing the manuscript. KK was involved in the study design, data interpretation and critical review of the manuscript. SD was involved in study design and management, data interpretation and manuscript preparation. WM helped conceive the study, and participated in its design and coordination, assisted with interpretation of the study results and helped to draft and review the manuscript. All authors read and approved the final manuscript.

## Pre-publication history

The pre-publication history for this paper can be accessed here:

http://www.biomedcentral.com/1471-244X/12/228/prepub

## References

[B1] Berk M Dodd S Malhi GS 'Bipolar missed states': the diagnosis and clinical salience of bipolar mixed states Aust N Z J Psychiatry 2005 39 215 221 15777356 10.1080/j.1440-1614.2004.01557.x

[B2] Merikangas KR Akiskal HS Angst J Greenberg PE Hirschfeld RM Petukhova M Kessler RC Lifetime and 12-month prevalence of bipolar spectrum disorder in the National Comorbidity Survey replication Arch Gen Psychiatry 2007 64 543 552 10.1001/archpsyc.64.5.543 17485606 PMC1931566

[B3] Goldney RD Fisher LJ Grande ED Taylor AW Hawthorne G Bipolar I and II disorders in a random and representative Australian population Aust N Z J Psychiatry 2005 39 726 729 10.1080/j.1440-1614.2005.01657.x 16050927

[B4] Zutshi A Eckert KA Hawthorne G Taylor AW Goldney RD Changes in the prevalence of bipolar disorders between 1998 and 2008 in an Australian population Bipolar Disord 2011 13 182 188 10.1111/j.1399-5618.2011.00907.x 21443572

[B5] Murray CJ Lopez AD The Global Burden of Disease Study 1996 Cambridge: Harvard University Press 10.1126/science.274.5288.740 8966556

[B6] Buckley PF Update on the treatment and management of schizophrenia and bipolar disorder CNS Spectr 2008 13 1 10 18227747 10.1017/s1092852900028212

[B7] Sachs GS Unmet clinical needs in bipolar disorder J Clin Psychopharmacol 2003 23 S2 S8 12832943 10.1097/01.jcp.0000084038.22282.47

[B8] Malhi GS Adams D Berk M Is lithium in a class of its own? A brief profile of its clinical use Aust N Z J Psychiatry 2009 43 1096 1104 10.3109/00048670903279937 20001408

[B9] Yatham LN Kennedy SH Schaffer A Parikh SV Beaulieu S O'Donovan C MacQueen G McIntyre RS Sharma V Ravindran A Young LT Young AH Alda M Milev R Vieta E Calabrese JR Berk M Ha K Kapczinski F Canadian Network for Mood and Anxiety Treatments (CANMAT) and International Society for Bipolar Disorders (ISBD) collaborative update of CANMAT guidelines for the management of patients with bipolar disorder: update 2009 Bipolar Disord 2009 11 225 255 10.1111/j.1399-5618.2009.00672.x 19419382

[B10] National Institute for Health and Clinical Excellence Guidance [Internet]. Bipolar Disorder: the management of bipolar disorder in adults, children and adolescents, in primary and secondary care 2006 London: National Institute for Health and Clinical Excellence (UK) Available from: http://www.ncbi.nlm.nih.gov/books/NBK11822/

[B11] Cipriani A Barbui C Salanti G Rendell J Brown R Stockton S Purgato M Spineli LM Goodwin GM Geddes JR Comparative efficacy and acceptability of antimanic drugs in acute mania: a multiple-treatments meta-analysis Lancet 2011 378 1306 1315 10.1016/S0140-6736(11)60873-8 21851976

[B12] Freeman MP Stoll AL Mood stabilizer combinations: a review of safety and efficacy Am J Psychiatry 1998 155 12 21 9433333 10.1176/ajp.155.1.12

[B13] Malhi GS Berk M Pharmacotherapy of bipolar disorder: the role of atypical antipsychotics and experimental strategies Hum Psychopharmacol 2002 17 407 412 10.1002/hup.437 12457376

[B14] Malhi GS Adams D Berk M Medicating mood with maintenance in mind: bipolar depression pharmacotherapy Bipolar Disord 2009 11 Suppl 2 55 76 19538686 10.1111/j.1399-5618.2009.00711.x

[B15] Hilty DM Leamon MH Lim RF Kelly RH Hales RE A review of bipolar disorder in adults Psychiatry 2006 3 43 55 20975827 PMC2963467

[B16] Baldessarini RJ Perry R Pike J Factors associated with treatment nonadherence among US bipolar disorder patients Hum Psychopharmacol 2008 23 95 105 10.1002/hup.908 18058849

[B17] Rush AJ Crismon ML Kashner TM Toprac MG Carmody TJ Trivedi MH Suppes T Miller AL Biggs MM Shores-Wilson K Witte BP Shon SP Rago WV Altshuler KZ TMAP Research Group Texas Medication Algorithm Project, phase 3 (TMAP-3): rationale and study design J Clin Psychiatry 2003 64 357 369 10.4088/JCP.v64n0402 12716235

[B18] Kogan JN Otto MW Bauer MS Dennehy EB Miklowitz DJ Zhang HW Ketter T Rudorfer MV Wisniewski SR Thase ME Calabrese J Sachs GS STEP-BD Investigators Demographic and diagnostic characteristics of the first 1000 patients enrolled in the Systematic Treatment Enhancement Program for Bipolar Disorder (STEP-BD) Bipolar Disord 2004 6 460 469 10.1111/j.1399-5618.2004.00158.x 15541061

[B19] Goetz I Tohen M Reed C Lorenzo M Vieta E Functional impairment in patients with mania: baseline results of the EMBLEM study Bipolar Disord 2007 9 45 52 10.1111/j.1399-5618.2007.00325.x 17391349

[B20] Bauer MS Kirk GF Gavin C Williford WO Determinants of functional outcome and healthcare costs in bipolar disorder: a high-intensity follow-up study J Affect Disord 2001 65 231 241 10.1016/S0165-0327(00)00247-0 11511403

[B21] Biffin F Tahtalian S Filia K Fitzgerald PB De Castella AR Filia S Berk M Dodd S Callaly P Berk L Kelin K Smith M Montgomery W Kulkarni J The impact of age at onset of bipolar I disorder on functioning and clinical presentation Acta Neuropsychiatrica 2009 21 191 196 10.1111/j.1601-5215.2009.00399.x 27397163

[B22] Kulkarni J Berk M Fitzgerald PB de Castella AR Montgomery W Kelin K Brnabic A Granger RE Dodd S The Bipolar Comprehensive Outcomes Study (BCOS): baseline findings of an Australian cohort study J Affect Disord 2008 107 135 144 10.1016/j.jad.2007.08.012 17889373

[B23] Berk M Dodd S Callaly P Berk L Fitzgerald P de Castella AR Filia S Filia K Tahtalian S Biffin F Kelin K Smith M Montgomery W Kulkarni J History of illness prior to a diagnosis of bipolar disorder or schizoaffective disorder J Affect Disord 2007 103 181 186 10.1016/j.jad.2007.01.027 17324469

[B24] Dodd S Kulkarni J Berk L Ng F Fitzgerald PB de Castella AR Filia S Filia K Montgomery W Kelin K Smith M Brnabic A Berk M A prospective study of the impact of subthreshold mixed states on the 24-month clinical outcomes of bipolar I disorder or schizoaffective disorder J Affect Disord 2010 124 22 28 10.1016/j.jad.2009.10.027 19944466

[B25] Abrams DJ Rojas DC Arciniegas DB Is schizoaffective disorder a distinct categorical diagnosis? A critical review of the literature Neuropsychiatr Dis Treat 2008 4 1089 1109 19337453 10.2147/ndt.s4120 PMC2646642

[B26] Schillevoort I de BA Herings RM Roos RA Jansen PA Leufkens HG Risk of extrapyramidal syndromes with haloperidol, risperidone, or olanzapine Ann Pharmacother 2001 35 1517 1522 11793611 10.1345/aph.1A068

[B27] Gomez JC Sacristan JA Hernandez J Breier A Ruiz CP Anton SC Fontova CE The safety of olanzapine compared with other antipsychotic drugs: results of an observational prospective study in patients with schizophrenia (EFESO Study). Pharmacoepidemiologic Study of Olanzapine in Schizophrenia J Clin Psychiatry 2000 61 335 343 10.4088/JCP.v61n0503 10847307

[B28] Collaborative Working Group on Clinical Trial Evaluations Clinical development of atypical antipsychotics: research design and evaluation J Clin Psychiatry 1998 59 Suppl 12 10 16 9766614

[B29] Malhi GS Green M Fagiolini A Peselow ED Kumari V Schizoaffective disorder: diagnostic issues and future recommendations Bipolar Disord 2008 10 215 230 10.1111/j.1399-5618.2007.00564.x 18199238

[B30] Benson K Hartz AJ A comparison of observational studies and randomized, controlled trials N Engl J Med 2000 342 1878 1886 10.1056/NEJM200006223422506 10861324

[B31] American Psychiatric Association Diagnostic and Statistical Manual of Mental Disorders Text Revision: DSM-IV-TR 2000 Washington, DC: American Psychiatry Press

[B32] Sheehan DV Lecrubier Y Sheehan KH Amorim P Janavs J Weiller E Hergueta T Baker R Dunbar GC The Mini-International Neuropsychiatric Interview (M.I.N.I.): the development and validation of a structured diagnostic psychiatric interview for DSM-IV and ICD-10 J Clin Psychiatry 1998 59 Suppl 20 22 33 9881538

[B33] Spearing MK Post RM Leverich GS Brandt D Nolen W Modification of the Clinical Global Impressions (CGI) Scale for use in bipolar illness (BP): the CGI-BP Psychiatry Res 1997 73 159 171 10.1016/S0165-1781(97)00123-6 9481807

[B34] Young RC Biggs JT Ziegler VE Meyer DA A rating scale for mania: reliability, validity and sensitivity Br J Psychiatry 1978 133 429 435 10.1192/bjp.133.5.429 728692

[B35] Hamilton M A rating scale for depression J Neurol Neurosurg Psychiatry 1960 23 56 62 10.1136/jnnp.23.1.56 14399272 PMC495331

[B36] Lam RW Michalak EE Swinson RP Assessment Scales in Depression, Mania and Anxiety 2005 London and New York: Taylor and Francis

[B37] Tohen M Frank E Bowden CL Colom F Ghaemi SN Yatham LN Malhi GS Calabrese JR Nolen WA Vieta E Kapczinski F Goodwin GM Suppes T Sachs GS Chengappa KR Grunze H Mitchell PB Kanba S Berk M The International Society for Bipolar Disorders (ISBD) Task Force report on the nomenclature of course and outcome in bipolar disorders Bipolar Disord 2009 11 453 473 10.1111/j.1399-5618.2009.00726.x 19624385

[B38] Tohen M Stoll AL Strakowski SM Foedda GL Mayer PV Goodwin DS Kolbrener ML Madigan AM The McLean first episode psychosis project: six-month recovery and recurrence outcome Schizophrenia Bull 1992 18 273 282 10.1093/schbul/18.2.273 1621073

[B39] The EuroQol Group EuroQol-a new facility for the measurement of health-related quality of life Health Policy 1990 16 199 208 10109801 10.1016/0168-8510(90)90421-9

[B40] Ware JE Jr Sherbourne CD The MOS 36-item short-form health survey (SF-36). I. Conceptual framework and item selection Med Care 1992 30 473 483 10.1097/00005650-199206000-00002 1593914

[B41] Keller MB Lavori PW Friedman B Nielsen E Endicott J McDonald-Scott P Andreasen NC The Longitudinal Interval Follow-up Evaluation. A comprehensive method for assessing outcome in prospective longitudinal studies Arch Gen Psychiatry 1987 44 540 548 10.1001/archpsyc.1987.01800180050009 3579500

[B42] Jablensky A McGrath J Herman H Castle D Gureje O Morgan V Korten A on behalf of the Low Prevalence Disorders Study Group People living with psychotic illness: an Australian Study National Surv Ment Health Wellbeing Rep 1999 4 1 22

[B43] World Health Organization Collaborating Centre for Drug Statistics Methodologyhttp://www.whocc.no/atcddd/indexdatabase/index.php

[B44] D'Agostino RB Jr Propensity score methods for bias reduction in the comparison of a treatment to a non-randomized control group Stat Med 1998 17 2265 2281 10.1002/(SICI)1097-0258(19981015)17:19<2265::AID-SIM918>3.0.CO;2-B 9802183

[B45] Rosenbaum PR Rubin DB The central role of the propensity score in observational studies for causal effects Biometrika 1983 70 41 55 10.1093/biomet/70.1.41

[B46] Simon J Pilling S Burbeck R Goldberg D Treatment options in moderate and severe depression: decision analysis supporting a clinical guideline Br J Psychiatry 2006 189 494 501 10.1192/bjp.bp.105.014571 17139032

[B47] Labour Force, Australia, Dec 2005 http://www.abs.gov.au/AUSSTATS/abs@.nsf/allprimarymainfeatures/42C56B422C8DACF4CA25701A0079FFDA?opendocument

[B48] Judd LL Akiskal HS Schettler PJ Endicott J Maser J Solomon DA Leon AC Rice JA Keller MB The long-term natural history of the weekly symptomatic status of bipolar I disorder Arch Gen Psychiatry 2002 59 530 537 10.1001/archpsyc.59.6.530 12044195

[B49] Baldessarini R Henk H Sklar A Chang J Leahy L Psychotropic medications for patients with bipolar disorder in the United States: polytherapy and adherence Psychiatr Serv 2008 59 1175 1183 10.1176/appi.ps.59.10.1175 18832504

[B50] Williams LJ Brennan SL Henry MJ Berk M Jacka FN Nicholson GC Kotowicz MA Pasco JA Area-based socioeconomic status and mood disorders: cross-sectional evidence from a cohort of randomly selected adult women Maturitas 2011 69 173 178 10.1016/j.maturitas.2011.03.015 21514078

[B51] Berk M Malhi GS Should antipsychotics take pole position in mania treatment? Lancet 2011 378 1279 1281 10.1016/S0140-6736(11)61060-X 21851975

